# Supplementary material for: Scaling laws of bacterial and archaeal plasmids
Source: Nat Commun. 2025 Jul 2;16:6023. doi: 10.1038/s41467-025-61205-2 (PMC12222811; doi:10.1038/s41467-025-61205-2)

SUPPLEMENTARY INFORMATION

Scaling laws of bacterial and archaeal plasmids

Rohan Maddamsetti[1,2,3,*], Irida Shyti[1,2], Maggie L. Wilson[1,2], Hye-In Son[1,2], Yasa Baig[4], Zhengqing Zhou[1,2], Jia Lu[1,2], Lingchong You[1,2,5,*]

[1]Center for Quantitative Biodesign, Duke University, Durham, NC, USA

[2]Department of Biomedical Engineering, Duke University, Durham, NC, USA.

[3]Department of Biochemistry and Microbiology, Rutgers University, New Brunswick, NJ, USA

[4]Department of Bioengineering, Stanford University, Stanford, CA, USA

[5]Department of Molecular Genetics and Microbiology, Duke University School of Medicine, Durham, NC, USA

*e-mail: rohan.maddamsetti@rutgers.edu, lingchong.you@duke.edu

Supplementary Table 1. Three out of fifty randomly chosen genomes containing plasmids have publications with reported plasmid copy numbers (PCN).

Genome Accession	Plasmid copy number published	PubMed ID
GCF_000145845.2_ASM14584v2	No	35250942
GCF_001690035.1_ASM169003v1	No	26769926
GCF_002073615.2_ASM207361v2	No	31346170
GCF_003146705.1_ASM314670v1	No	30910889
GCF_003193665.1_ASM319366v1	No	29118174
GCF_003345315.1_ASM334531v1	No	30533938
GCF_003812945.1_ASM381294v1	No	31346170
GCF_004768665.1_ASM476866v1	No	31738764
GCF_004798785.1_ASM479878v1	No	31738764
GCF_009892325.1_ASM989232v1	No	32670207 and 32625185
GCF_010365345.1_ASM1036534v1	No	32806462
GCF_011331065.1_ASM1133106v1	No	33164081
GCF_013167675.1_ASM1316767v1	No	34751643
GCF_013589875.1_ASM1358987v1	No	34609275
GCF_013590775.1_ASM1359077v1	No	34609275
GCF_013728275.1_ASM1372827v1	No	34609275
GCF_013740875.1_ASM1374087v1	No	34609275
GCF_014131795.1_ASM1413179v1	No	NA
GCF_014268495.2_ASM1426849v2	No	34442845
GCF_015475575.1_ASM1547557v1	No	34596508
GCF_016904115.1_ASM1690411v1	No	31346170
GCF_017165275.1_ASM1716527v1	No	34751643
GCF_017639045.1_ASM1763904v1	No	33842964
GCF_017639065.1_ASM1763906v1	No	33842964

GCF_017639165.1_ASM1763916v1	No	33842964
GCF_018075145.1_ASM1807514v1	No	35379360
GCF_018884145.1_ASM1888414v1	No	NA
GCF_018885165.1_ASM1888516v1	No	34748382
GCF_019048185.1_ASM1904818v1	No	31346170
GCF_019551835.1_ASM1955183v1	No	34900963
GCF_020149825.1_ASM2014982v1	**Yes**	35416146
GCF_021513055.1_ASM2151305v1	No	NA
GCF_022494825.1_ASM2249482v1	No	35909619
GCF_022494855.1_ASM2249485v1	No	35909619
GCF_022925255.1_ASM2292525v1	No	NA
GCF_023278615.1_ASM2327861v1	No	32328058
GCF_024266205.1_ASM2426620v1	No	31383653
GCF_024508075.1_ASM2450807v1	No	NA
GCF_024918255.1_ASM2491825v1	**Yes**	36036505
GCF_025349945.1_ASM2534994v1	**Yes**	36314936
GCF_027944875.1_ASM2794487v1	No	36916926
GCF_027945055.1_ASM2794505v1	No	36916926
GCF_028335185.1_ASM2833518v1	No	37389336
GCF_029873515.1_ASM2987351v1	No	NA
GCF_030037835.1_ASM3003783v1	No	37659628
GCF_030038915.1_ASM3003891v1	No	37659628
GCF_900175995.1_ASM90017599v1	No	28433722
GCF_900620255.1_BPH2986	No	36934086
GCF_903993065.2_AI3007v1_cp	No	38010338
GCF_905071835.1_MSB1_4I	No	NA

Supplementary Table 2. Simple performance comparison between pseuPIRA and CoverM

RefSeq ID	SRA ID	FASTQ data (Gb)	Software	Run time* (seconds)	Plasmid copy numbers (per chromosome)
GCF_013742375.1	SRR11948691	1.58	pseuPIRA (quick mode)	16.31 s	1.2, 1.9, 1.6, 23, 45, 27, 29, 6.2, 20.7
GCF_013742375.1	SRR11948691	1.58	pseuPIRA (full mode)	16.61 s	1.3, 2.0, 1.8, 23, 45, 27, 30, 6.2, 20.7
GCF_013742375.1	SRR11948691	1.58	CoverM	12.90 s	1.4, 2.1, 2.0, 23, 45, 27, 29, 6.2, 20.6
GCF_002285515.1	SRR1974308	90.6	pseuPIRA (quick mode)	688.87 s	2.2
GCF_002285515.1	SRR1974308	90.6	pseuPIRA (full mode)	705.04 s	2.3
GCF_002285515.1	SRR1974308	90.6	CoverM	1175.65 s	2.3

*Real time reported by the /usr/bin/time command-line utility on a Macbook Pro M1 laptop, with 4 cores allocated for each program run.

Supplementary Table 3. Details of segmented regression models.

Regression formula*	β_0	β_1	β_2	ψ
$\log_{10}(\text{copy number}) \sim \beta_0 + \beta_1(\log_{10}(\text{length})) + \beta_2(\log_{10}(\text{length}) - \psi) \times I(\log_{10}(\text{length}) > \psi)$	4.6904	–0.9577	0.8027	4.753
$\log_{10}(\text{copy number}) \sim \beta_0 + \beta_1(\log_{10}(\text{normalized length})) + \beta_2(\log_{10}(\text{normalized length}) - \psi) \times I(\log_{10}(\text{normalized length}) > \psi)$	–1.4292	–0.8787	0.7534	–1.735

* Here, $I(\cdot)$ is the indicator function equal to one when the statement is true. Here, β_1 is the left slope of the segmented regression, β_2 is the difference-in-slopes and ψ is the breakpoint. See Muggeo[39] for further details about this parameterization. The second model uses plasmid length normalized by the length of the largest chromosome in the genome.

Supplementary Figure 1. Plasmid copy number estimation benchmarking. Plasmids with PIRA PCN < 0.8 are labeled in red. In each panel, a linear regression showing the observed line of best fit is drawn in light blue. A dashed red line indicates the expected one-to-one correlation. These results indicate that PIRA provides accurate and reliable PCN estimates and show that the low PCN estimates (PCN < 0.8) are a property of the underlying sequencing data, given the consistency of these estimates across methods.

A) Comparison of direct Themisto PCN estimates to PIRA PCN estimates, including plasmids with fewer than 10,000 mapped reads by the direct method (shown as triangles), shows that PIRA recovers more PCN estimates through the inclusion of multiread data. Pearson correlation $\rho = 0.959$.

B) Comparison of direct Themisto PCN estimates to PIRA PCN estimates, excluding plasmids with fewer than 10,000 mapped reads by the direct method. Pearson correlation $\rho = 0.997$.

C) Benchmarking of PIRA against minimap2. PCN estimates were compared between PIRA and minimap2 on a test set of 100 randomly selected genomes, each with at least one plasmid with PIRA PCN < 0.8. Pearson correlation $\rho = 0.998$.

D) Benchmarking of PIRA against *breseq*. PCN estimates were compared between PIRA and *breseq* on a test set of 100 randomly selected genomes, each with at least one plasmid with PIRA PCN < 0.8. Pearson correlation $\rho = 0.991$.

E) Comparison of direct *themisto* PCN estimates to direct *kallisto* PCN estimates. Pearson correlation $\rho = 0.991$.

F) Comparison of PIRA PCN estimates against previously published PCN estimates in Supplementary Table 2 of Shaw et al.[13]. Pearson correlation $\rho = 0.997$.

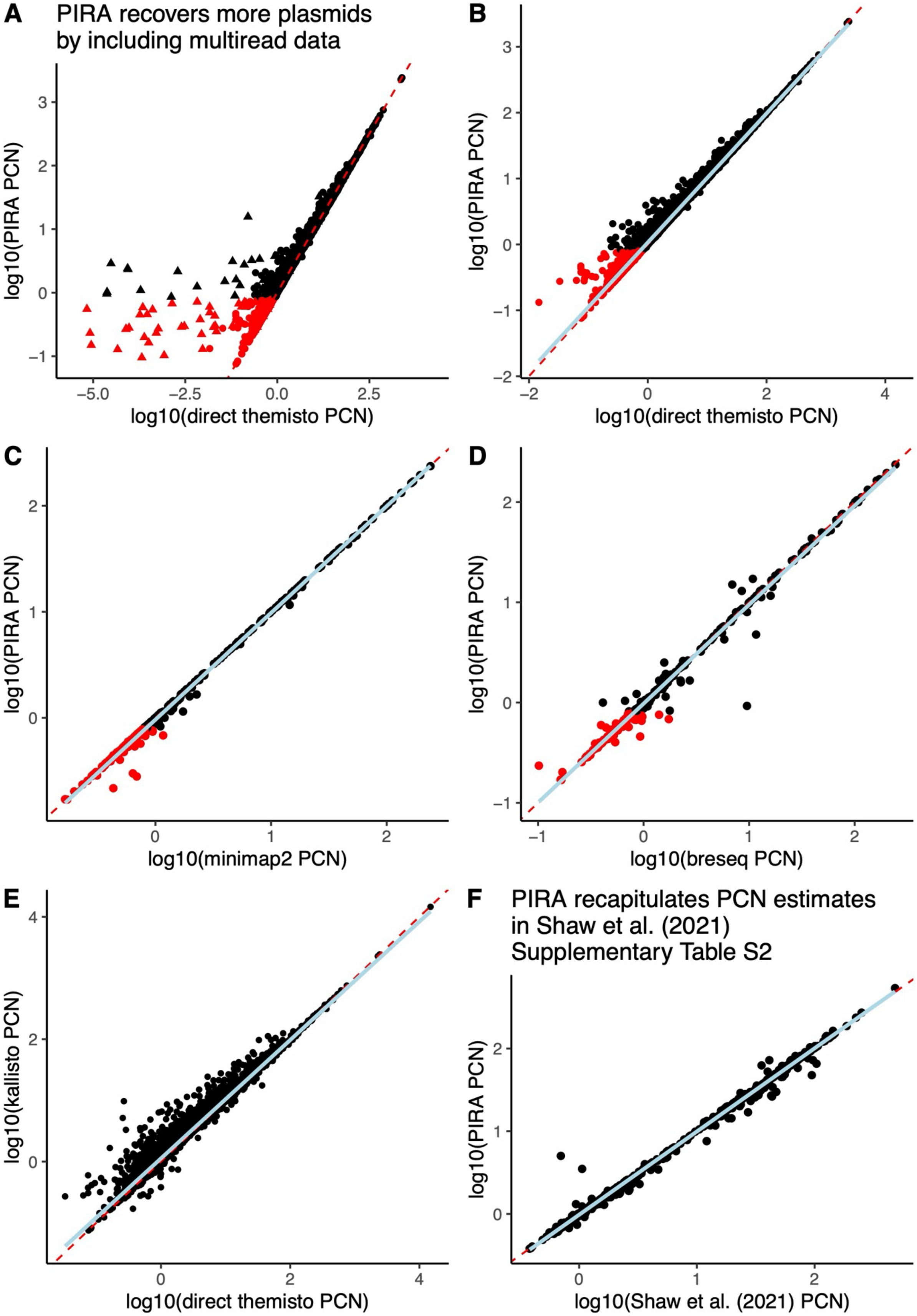
A
PIRA recovers more plasmids
by including multiread data
log10(PIRA PCN)
log10(direct themisto PCN)
B
log10(PIRA PCN)
log10(direct themisto PCN)
C
log10(PIRA PCN)
log10(minimap2 PCN)
D
log10(PIRA PCN)
log10(breseq PCN)
E
log10(kallisto PCN)
log10(direct themisto PCN)
F
PIRA recapitulates PCN estimates
in Shaw et al. (2021)
Supplementary Table S2
log10(PIRA PCN)
log10(Shaw et al. (2021) PCN)

Supplementary Figure 2. Summary statistics for plasmids binned by percentiles by length. Normally distributed 95% confidence intervals around the mean for each percentile by length are in black. The mean PCN for each percentile by length are in red, and the Q25 and Q75 PCN quantiles for the percentiles by length are in blue.

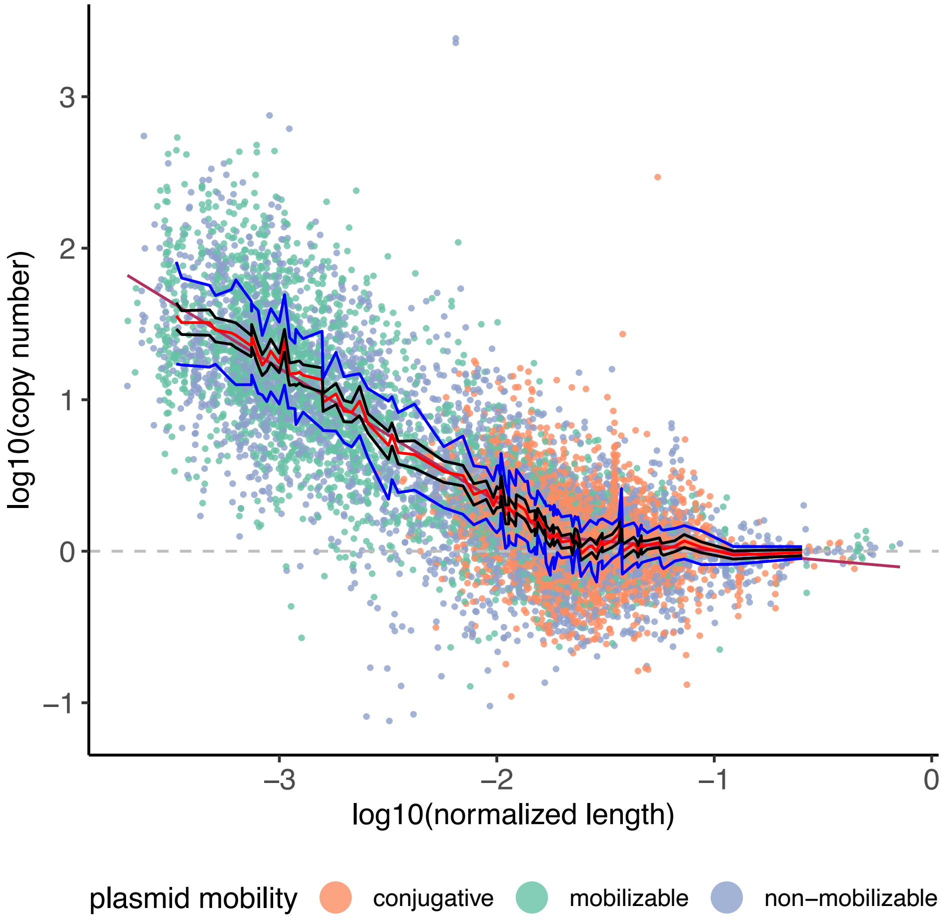

70

Supplementary Figure 3. Plasmid length inversely correlates with plasmid copy number.

A) Plasmid length inversely correlates with plasmid copy number. Even without rescaling plasmid length by the length of the largest chromosome, as in Figure 1, a biphasic scaling law is apparent. A segmented regression (in maroon) was fit to these data on a log-log plot. This segmented regression has a first slope of –0.96, a breakpoint at 4.69, a second slope of –0.16, and an Adjusted R^2 of 0.692. The marginal density distributions of plasmid copy number and length are displayed on the axes.

B) The inverse correlation between plasmid length and copy number holds across diverse environments.

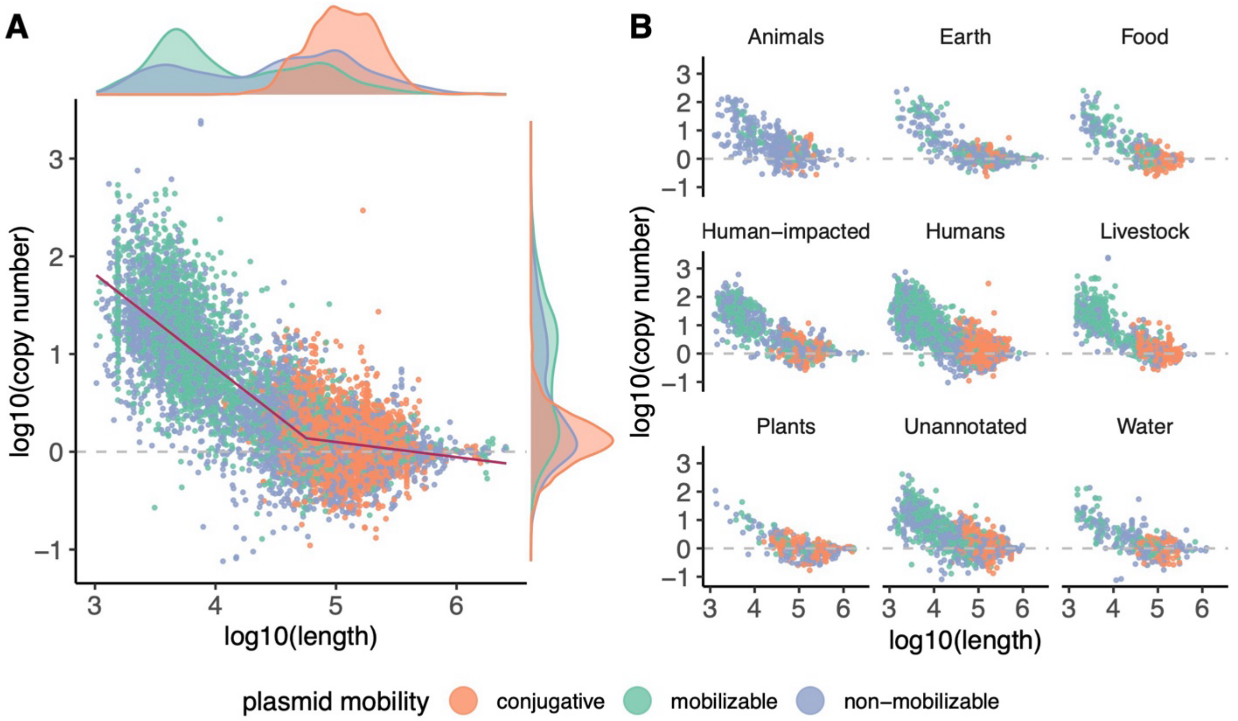

Supplementary Figure 4. The inverse correlation between plasmid copy number and plasmid length holds across genera. Conjugative plasmids are colored orange pink, mobilizable plasmids are colored light green, and non-mobilizable plasmids are colored light blue.

A) Each panel represents a NCBI taxonomic group with at least 50 plasmids; genera with fewer than 50 plasmids are lumped together in the final panel.

B) Each panel represents a NCBI taxonomic subgroup with at least 50 plasmids; genera with fewer than 50 plasmids are lumped together in the final panel.

C) Each panel represents a genus with at least 50 plasmids; genera with fewer than 50 plasmids are lumped together in the final panel.

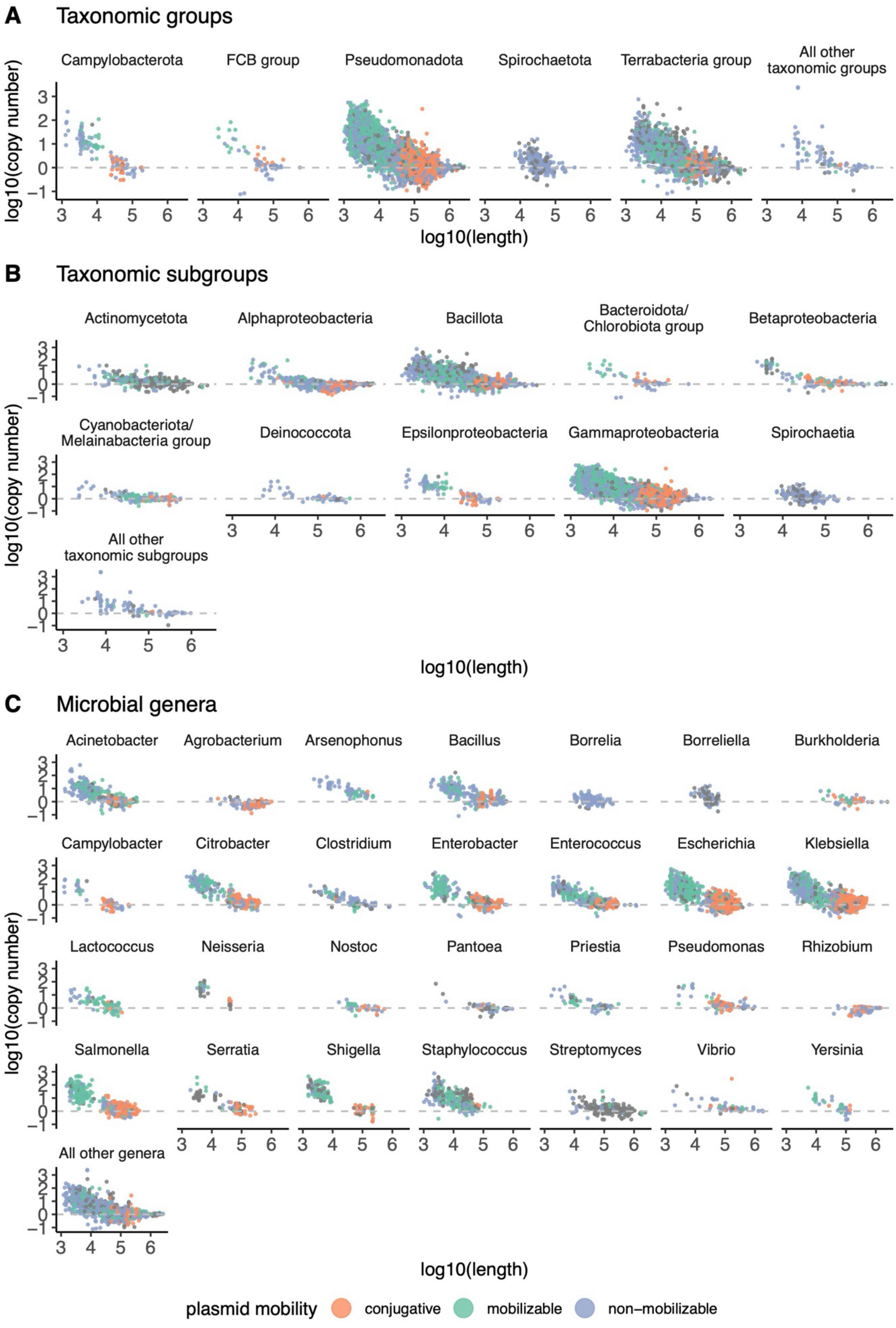
A Taxonomic groups
Campylobacterota
FCB group
Pseudomonadota
Spirochaetota
Terrabacteria group
All other taxonomic groups
log10(copy number)
log10(length)
B Taxonomic subgroups
Actinomycetota
Alphaproteobacteria
Bacillota
Bacteroidota/ Chlorobiota group
Betaproteobacteria
Cyanobacteriota/ Melainabacteria group
Deinococcota
Epsilonproteobacteria
Gammaproteobacteria
Spirochaetia
All other taxonomic subgroups
log10(copy number)
log10(length)
C Microbial genera
Acinetobacter
Agrobacterium
Arsenophonus
Bacillus
Borrelia
Borreliella
Burkholderia
Campylobacter
Citrobacter
Clostridium
Enterobacter
Enterococcus
Escherichia
Klebsiella
Lactococcus
Neisseria
Nostoc
Pantoea
Priestia
Pseudomonas
Rhizobium
Salmonella
Serratia
Shigella
Staphylococcus
Streptomyces
Vibrio
Yersinia
All other genera
log10(copy number)
log10(length)
plasmid mobility
conjugative
mobilizable
non-mobilizable

Supplementary Figure 5. The inverse power-law between plasmid length and PCN holds across plasmid taxonomic units (PTUs). Conjugative plasmids are colored orange pink, mobilizable plasmids are colored light green, and non-mobilizable plasmids are colored light blue. Each row represents a plasmid classification scheme described by different groups, to show the robustness of this finding to the choice of classification method. In every panel, PTUs are ranked by length from smallest to largest, to show variation within PTUs. The left panel shows variation in plasmid lengths within PTUs (i.e., per each rank on the x-axis). The right panel shows variation in plasmid copy numbers within PTUs (i.e., per each rank on the x-axis).

A) Plasmid taxonomic units (PTUs) defined by the similarity network analysis by Acman et al. (2020) cluster by length.

B) PTUs defined by the similarity network analysis by Acman et al. (2020) vary more in copy number than length.

C) The inverse correlation between length and copy number holds across PTUs defined by Acman et al. (2020).

D) PTUs defined by the similarity network analysis by Redondo-Salvo et al. (2020) cluster by length.

E) PTUs defined by the similarity network analysis by Redondo-Salvo et al. (2020) vary more in copy number than length.

F) The inverse correlation between length and copy number holds across PTUs defined by Redondo-Salvo et al. (2020).

G) PTUs defined by MOB-Cluster cluster by length.

H) PTUs defined by MOB-Cluster vary more in copy number than length.

I) The inverse correlation between length and copy number holds across PTUs defined by MOB-Cluster.

J) PTUs defined by MOB-Typer Rep protein typing cluster by length.

K) PTUs defined by MOB-Typer Rep protein typing vary more in copy number than length.

L) The inverse correlation between length and copy number holds across PTUs defined by MOB-Typer.

M) PTUs defined by the Rep protein typing in Ares-Arroyo et al. (2023) cluster by length.

N) PTUs defined by the Rep protein typing in Ares-Arroyo et al. (2023) vary more in copy number than length.

O) The inverse correlation between length and copy number holds across PTUs defined by Ares-Arroyo et al. (2023).

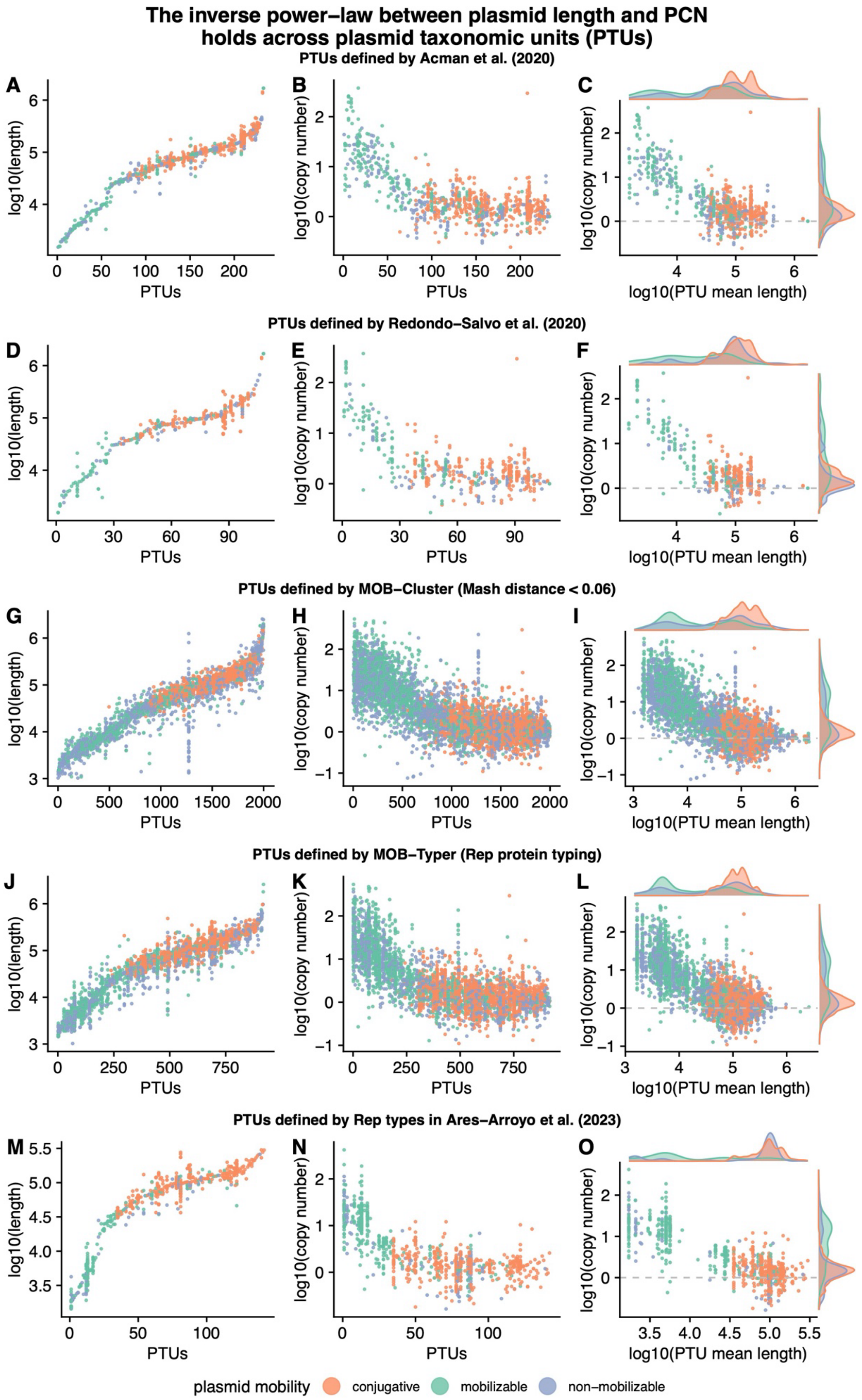
The inverse power–law between plasmid length and PCN
holds across plasmid taxonomic units (PTUs)
PTUs defined by Acman et al. (2020)
A
B
C
log10(length)
log10(copy number)
PTUs
log10(PTU mean length)
PTUs defined by Redondo–Salvo et al. (2020)
D
E
F
PTUs defined by MOB–Cluster (Mash distance < 0.06)
G
H
I
PTUs defined by MOB–Typer (Rep protein typing)
J
K
L
PTUs defined by Rep types in Ares–Arroyo et al. (2023)
M
N
O
plasmid mobility
conjugative
mobilizable
non–mobilizable

Supplementary Figure 6. Plasmid mobility groups defined by relaxase types with MOB-Typer often contain both small mobilizable plasmids and large conjugative plasmids. Conjugative plasmids are colored orange pink and mobilizable plasmids are colored light green. Relaxase types with more than 50 plasmids are shown in separate panels; the remaining relaxase types are lumped together in the final panel. Some plasmids have multiple relaxase enzymes and may therefore belong to multiple mobility groups. For instance, the MOBF,MOBF panel indicates plasmids that contain two MOBF-family relaxases, and the MOBF,MOBP panel indicates plasmids that contain one MOBF-family relaxase and one MOBP-family relaxase.

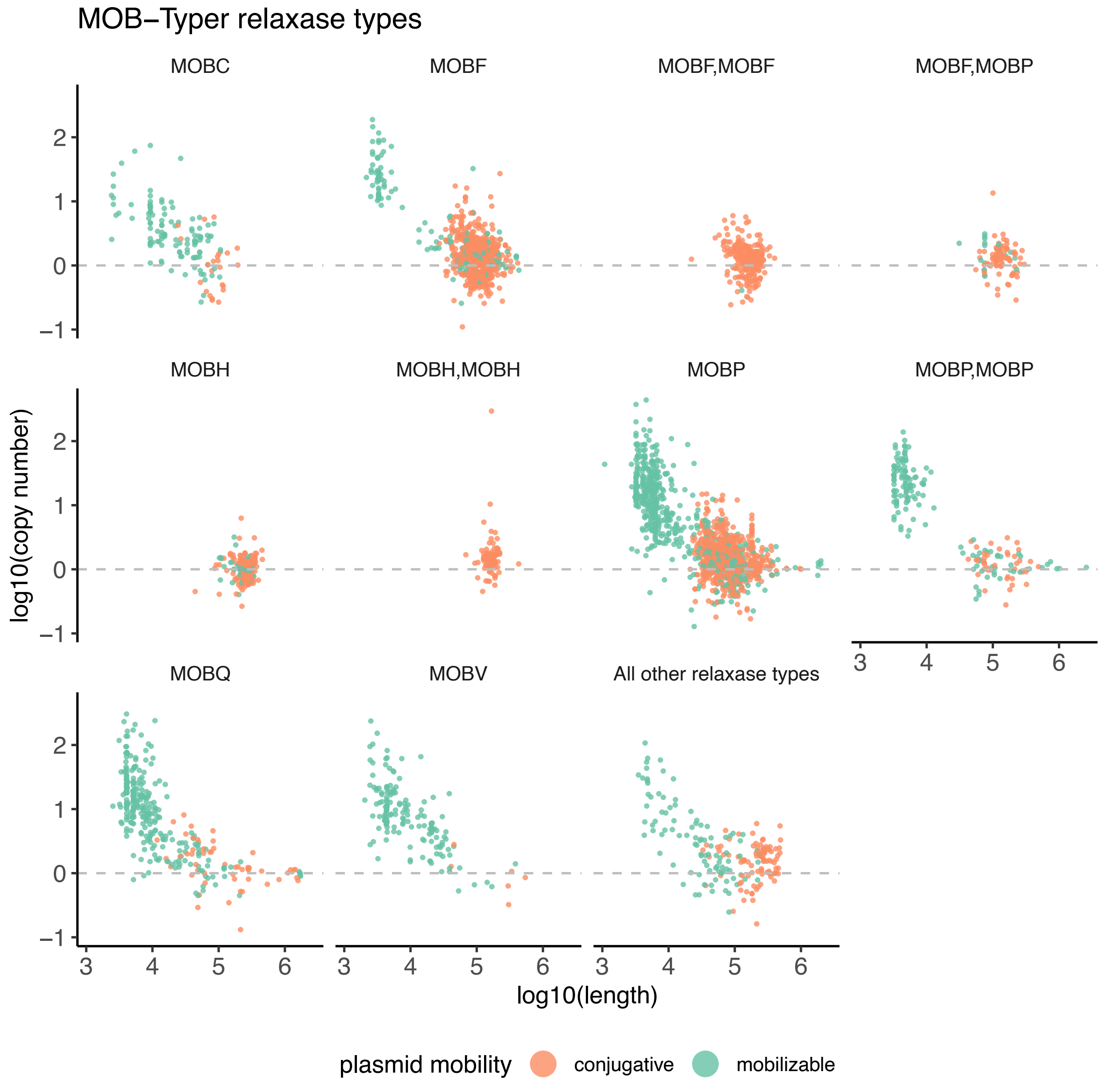

Supplementary Figure 7. Plasmid host range does not correlate with plasmid length or copy number. Conjugative plasmids are colored orange pink, mobilizable plasmids are colored light green, and non-mobilizable plasmids are colored light blue.

A) Host range annotated with MOB-Typer. Host ranges with more than 50 plasmids are shown in separate panels; the remaining host ranges are lumped together in the final panel.

B) Host range annotated by Redondo-Salvo et al. (2020). Host range are classified from I (most narrow host range) to VI (broadest host range).

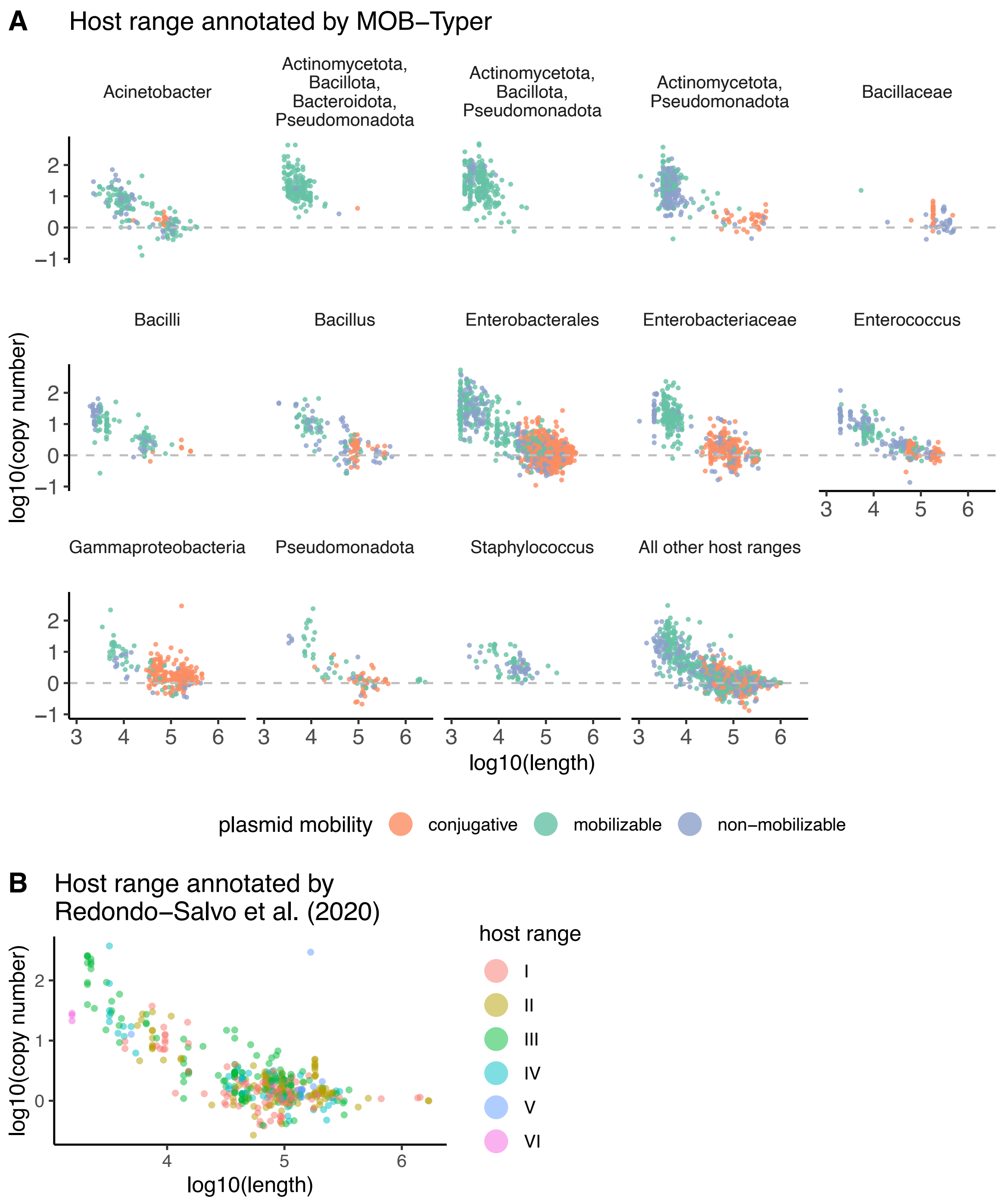

Supplementary Figure 8. Low-copy number plasmids are common, while high copy number plasmids are rare and are enriched in human-impacted environments.

A) Histogram of plasmid copy numbers across ecological categories. Dashed lines are drawn at PCN = 1 and PCN = 50.

B) Proportion of very low copy number (PCN < 1) plasmids per ecological category. Each point represents the proportion of isolates containing very low copy number plasmids (PCN < 1) within each ecological category. Error bars represent 95% binomial proportion confidence intervals around the mean, using the formula $p \pm Z_{\alpha/2}\sqrt(\frac{p(1-p)}{n})$, where *p* is the proportion, *n* is the sample size, and $Z_{\alpha/2}$ = 1.96.

C) Proportion of high copy number (PCN > 50) plasmids per ecological category. Each point represents the proportion of isolates containing high copy number plasmids (PCN > 50) within each ecological category. Error bars use the same formula as in panel B).

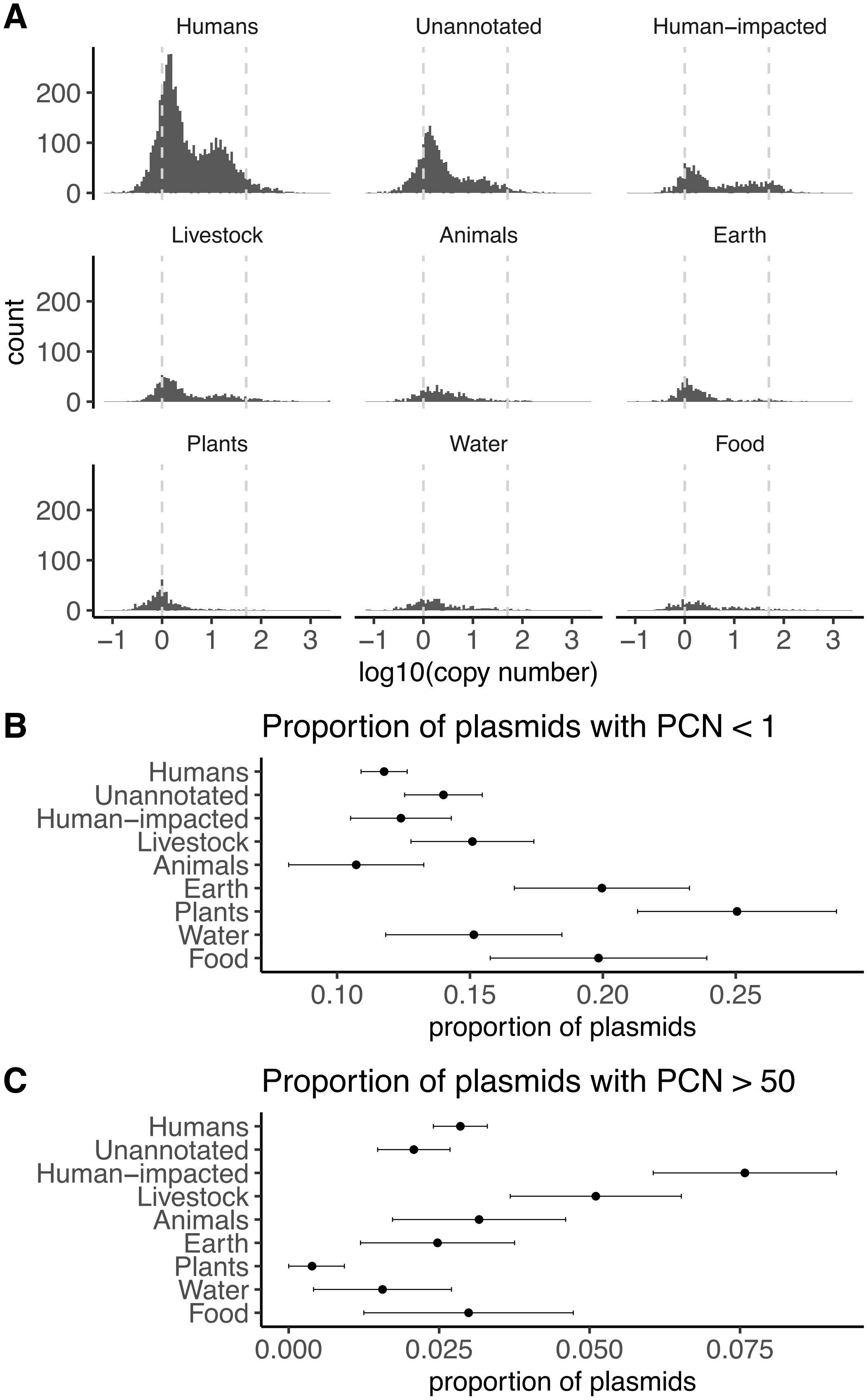
A
Humans
Unannotated
Human−impacted
Livestock
Animals
Earth
Plants
Water
Food
count
0
100
200
−1
0
1
2
3
log10(copy number)
B
Proportion of plasmids with PCN < 1
Humans
Unannotated
Human−impacted
Livestock
Animals
Earth
Plants
Water
Food
0.10
0.15
0.20
0.25
proportion of plasmids
C
Proportion of plasmids with PCN > 50
Humans
Unannotated
Human−impacted
Livestock
Animals
Earth
Plants
Water
Food
0.000
0.025
0.050
0.075
proportion of plasmids

Supplementary Figure 9. Protein-coding sequences on plasmids follow an empirical scaling law that holds across genera. Plasmids are shown in blue, megaplasmids (plasmid length > 500,000 bp) are shown in red, chromosomes are shown in green.

A) Each panel represents a NCBI taxonomic group with at least 50 plasmids; genera with fewer than 50 plasmids are lumped together in the final panel.

B) Each panel represents a NCBI taxonomic subgroup with at least 50 plasmids; genera with fewer than 50 plasmids are lumped together in the final panel.

C) Each panel represents a genus with at least 50 plasmids; genera with fewer than 50 plasmids are lumped together in the final panel.

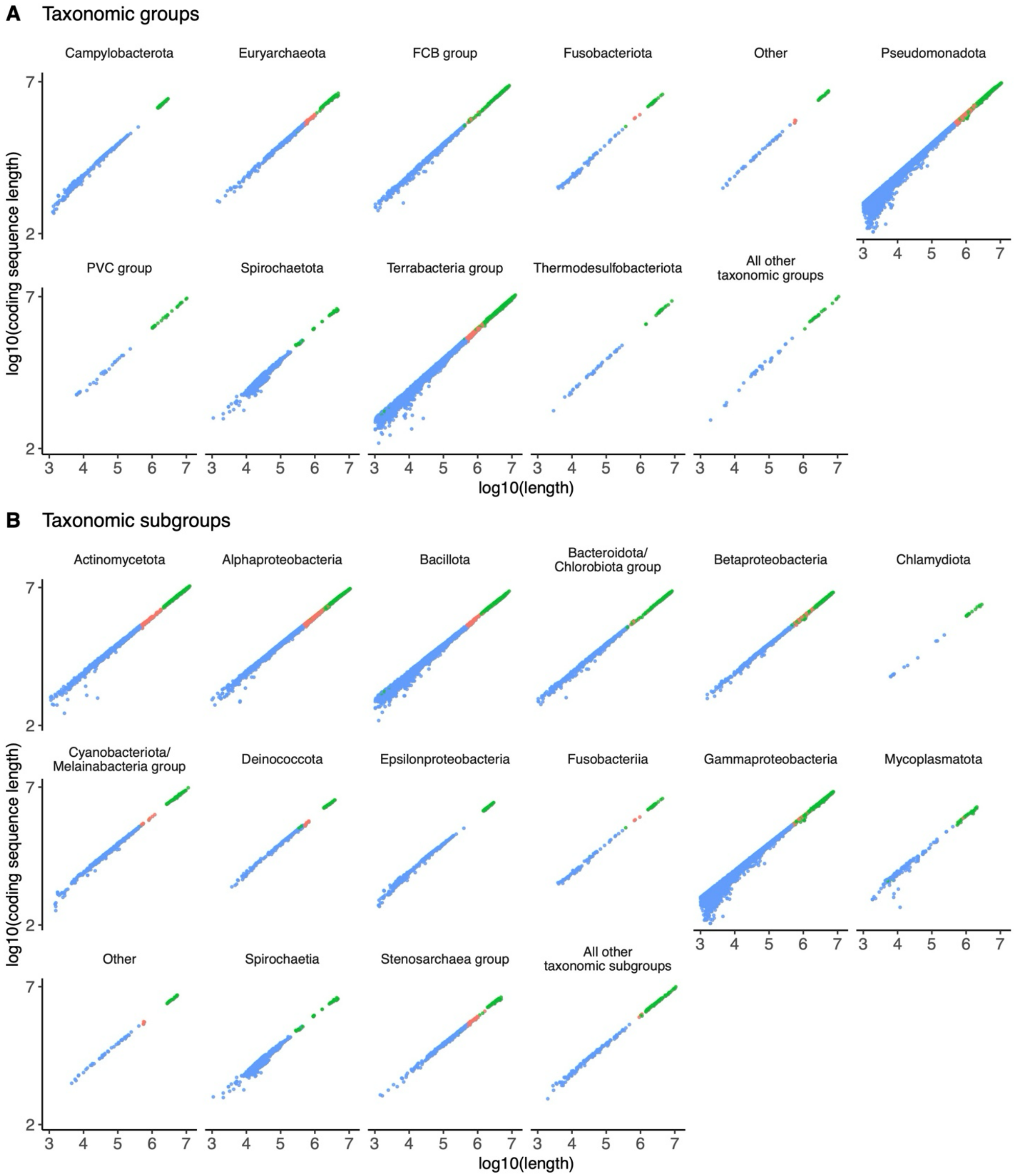
A Taxonomic groups
Campylobacterota
Euryarchaeota
FCB group
Fusobacteriota
Other
Pseudomonadota
PVC group
Spirochaetota
Terrabacteria group
Thermodesulfobacteriota
All other taxonomic groups
log10(coding sequence length)
log10(length)
B Taxonomic subgroups
Actinomycetota
Alphaproteobacteria
Bacillota
Bacteroidota/ Chlorobiota group
Betaproteobacteria
Chlamydiota
Cyanobacteriota/ Melainabacteria group
Deinococcota
Epsilonproteobacteria
Fusobacteriia
Gammaproteobacteria
Mycoplasmatota
Other
Spirochaetia
Stenosarchaea group
All other taxonomic subgroups
log10(coding sequence length)
log10(length)

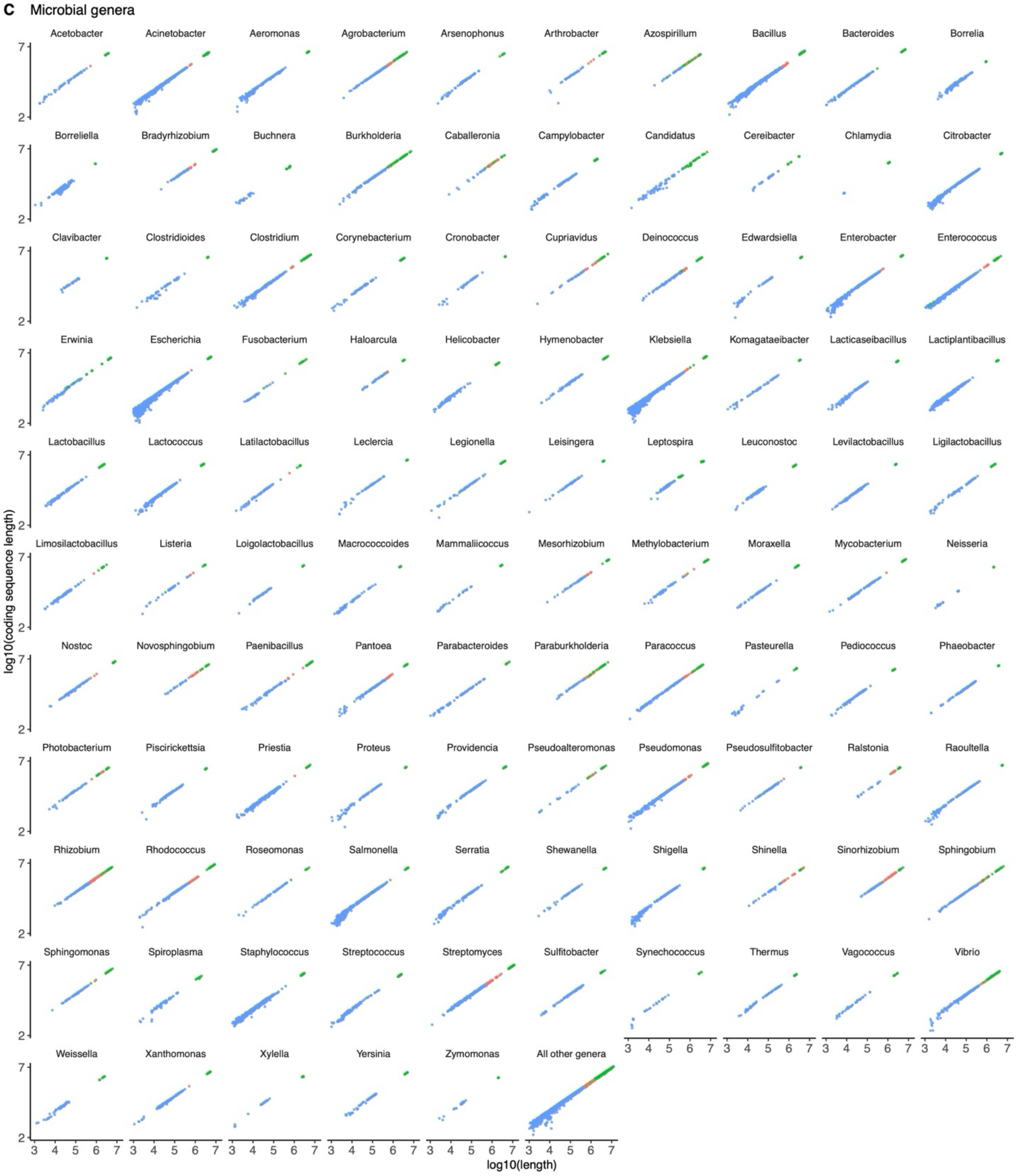
C Microbial genera
Acetobacter
Acinetobacter
Aeromonas
Agrobacterium
Arsenophonus
Arthrobacter
Azospirillum
Bacillus
Bacteroides
Borrelia
Borreliella
Bradyrhizobium
Buchnera
Burkholderia
Caballeronia
Campylobacter
Candidatus
Cereibacter
Chlamydia
Citrobacter
Clavibacter
Clostridioides
Clostridium
Corynebacterium
Cronobacter
Cupriavidus
Deinococcus
Edwardsiella
Enterobacter
Enterococcus
Erwinia
Escherichia
Fusobacterium
Haloarcula
Helicobacter
Hymenobacter
Klebsiella
Komagataeibacter
Lacticaseibacillus
Lactiplantibacillus
Lactobacillus
Lactococcus
Latilactobacillus
Leclercia
Legionella
Leisingera
Leptospira
Leuconostoc
Levilactobacillus
Ligilactobacillus
Limosilactobacillus
Listeria
Loigolactobacillus
Macrococcoides
Mammaliicoccus
Mesorhizobium
Methylobacterium
Moraxella
Mycobacterium
Neisseria
Nostoc
Novosphingobium
Paenibacillus
Pantoea
Parabacteroides
Paraburkholderia
Paracoccus
Pasteurella
Pediococcus
Phaeobacter
Photobacterium
Piscirickettsia
Priestia
Proteus
Providencia
Pseudoalteromonas
Pseudomonas
Pseudosulfitobacter
Ralstonia
Raoultella
Rhizobium
Rhodococcus
Roseomonas
Salmonella
Serratia
Shewanella
Shigella
Shinella
Sinorhizobium
Sphingobium
Sphingomonas
Spiroplasma
Staphylococcus
Streptococcus
Streptomyces
Sulfitobacter
Synechococcus
Thermus
Vagococcus
Vibrio
Weissella
Xanthomonas
Xylella
Yersinia
Zymomonas
All other genera
log10(coding sequence length)
log10(length)

Supplementary Figure 10. Metabolic genes on plasmids follow an empirical scaling law that holds across genera containing megaplasmids (plasmid length > 500,000 bp). Plasmids are shown in blue, megaplasmids (plasmids > 500,000 bp in length) are shown in red, chromosomes are shown in green. The linear regression between $\log_{10}$(metabolic genes) and $\log_{10}$(length) for chromosomes is shown in black.

A) Each panel represents a NCBI taxonomic group with at least 50 plasmids; genera with fewer than 50 plasmids are lumped together in the final panel.

B) Each panel represents a NCBI taxonomic subgroup with at least 50 plasmids; genera with fewer than 50 plasmids are lumped together in the final panel.

C) Each panel represents a genus with at least 50 plasmids; genera with fewer than 50 plasmids are lumped together in the final panel.

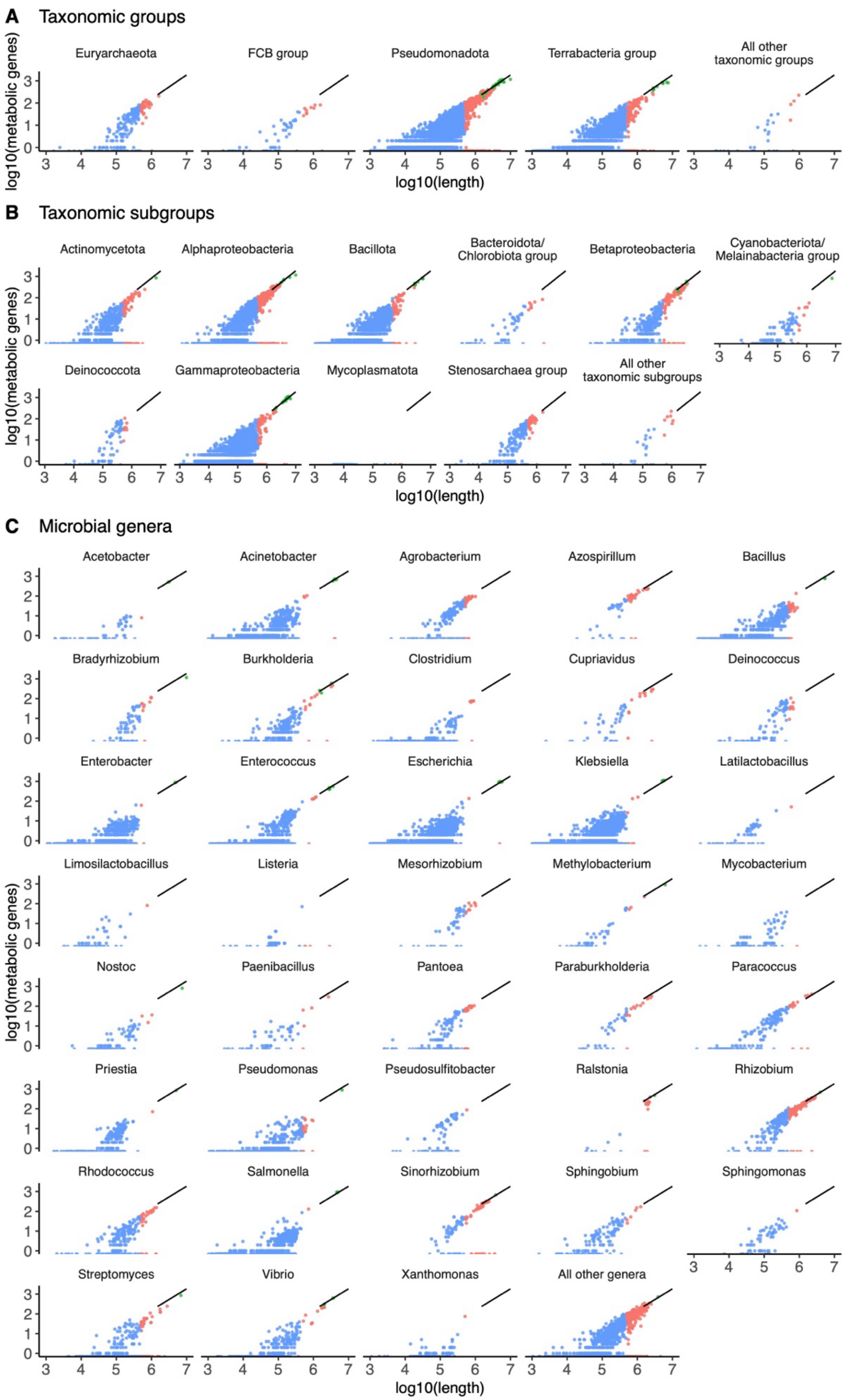

A Taxonomic groups
Euryarchaeota
FCB group
Pseudomonadota
Terrabacteria group
All other taxonomic groups
log10(metabolic genes)
log10(length)
B Taxonomic subgroups
Actinomycetota
Alphaproteobacteria
Bacillota
Bacteroidota/ Chlorobiota group
Betaproteobacteria
Cyanobacteriota/ Melainabacteria group
Deinococcota
Gammaproteobacteria
Mycoplasmatota
Stenosarchaea group
All other taxonomic subgroups
C Microbial genera
Acetobacter
Acinetobacter
Agrobacterium
Azospirillum
Bacillus
Bradyrhizobium
Burkholderia
Clostridium
Cupriavidus
Deinococcus
Enterobacter
Enterococcus
Escherichia
Klebsiella
Latilactobacillus
Limosilactobacillus
Listeria
Mesorhizobium
Methylobacterium
Mycobacterium
Nostoc
Paenibacillus
Pantoea
Paraburkholderia
Paracoccus
Priestia
Pseudomonas
Pseudosulfitobacter
Ralstonia
Rhizobium
Rhodococcus
Salmonella
Sinorhizobium
Sphingobium
Sphingomonas
Streptomyces
Vibrio
Xanthomonas
All other genera

Supplementary Figure 11. Plasmid DNA content (plasmid copy number × plasmid length) normalized by chromosome DNA content. A segmented regression (in maroon) was fit to these data on a log-log plot. This segmented regression has a first slope of 0.118, a breakpoint at –1.746, a second slope of 0.747, and an Adjusted R^2 of 0.316. The marginal density distributions of plasmid copy number and length are displayed on the axes.

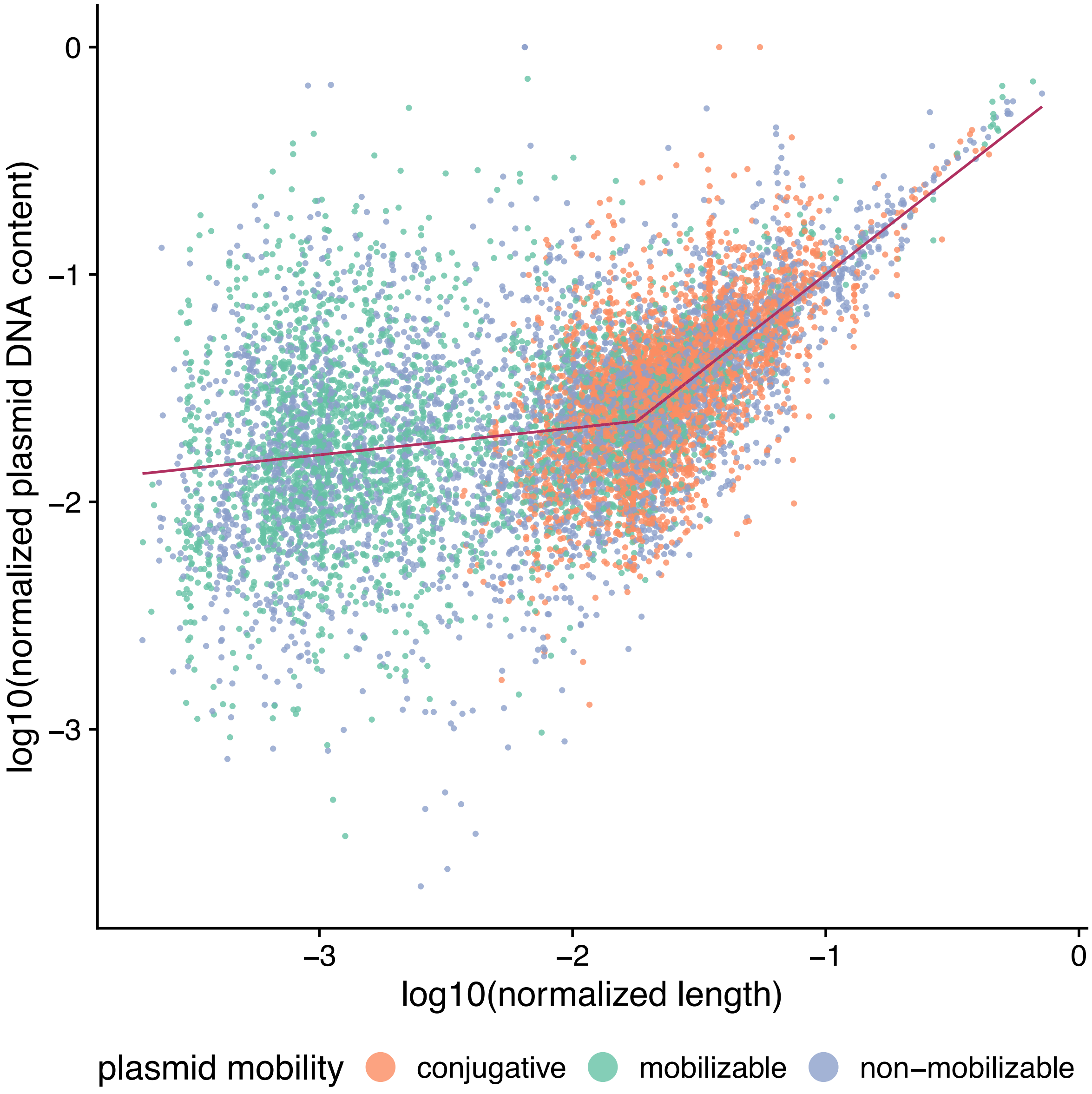

Supplement: Supplementary file 1 — Supplementary Information [file 41467_2025_61205_MOESM1_ESM.pdf]
