# Supplementary material for: Scaling laws of bacterial and archaeal plasmids
Source: Nat Commun. 2025 Jul 2;16:6023. doi: 10.1038/s41467-025-61205-2 (PMC12222811; doi:10.1038/s41467-025-61205-2)
Supplement: Supplementary file 2 — Description of Additional Supplementary Files [file 41467_2025_61205_MOESM2_ESM.pdf]

Description of Additional Supplementary Files:

Supplementary Data 1: CSV file of plasmid copy number estimates.

Supplementary Data 2: CSV file of PCN and length summary statistics for plasmid percentiles by length.

Supplementary Data 3: CSV file of PCN and length summary statistics for the two plasmid clusters.
